# Microalgae: A Promising Source of Bioactive Polysaccharides for Biotechnological Applications

**DOI:** 10.3390/molecules30092055

**Published:** 2025-05-05

**Authors:** Chiara Magnabosco, Giovanna Santaniello, Giovanna Romano

**Affiliations:** 1 National Research Council-Water Research Institute, Corso Tonolli 50, 28922 Verbania-Pallanza, Italy; chiaramagnabosco@cnr.it; 2 Science and High Technology Department, University of Insubria, via Valleggio 11, 22100 Como, Italy; 3 Stazione Zoologica Anton Dohrn, Ecosustainable Marine Biotechnology Department, via Acton 55, 80133 Naples, Italy; giovanna.santaniello@szn.it; 4 NBFC, National Biodiversity Future Center, 90133 Palermo, Italy

**Keywords:** polysaccharides, microalgae, bioactivity, cosmetic applications

## Abstract

Polysaccharides (PSs) are the most abundant carbohydrates in nature, performing essential biological functions such as immune system regulation, structural support, and cell communication. PSs from marine microalgae have gained increasing attention due to their diverse biological activities and potential applications in various fields, including the human health sector. These natural macromolecules, primarily composed of glucose, xylose, galactose, rhamnose, and fucose, exhibit bioactive properties influenced by their molecular weight, sulfation degree, and structural complexity. Microalgal PSs can function as antiviral, antimicrobial, antioxidant, immunomodulatory, and antitumor agents, making them promising candidates for pharmaceutical and nutraceutical applications. Additionally, their physicochemical properties make them valuable as bioactive ingredients in cosmetics, serving as hydrating agents, UV protectants, and anti-ageing compounds. The production of PSs from microalgae presents a sustainable alternative to terrestrial plants, as microalgae can be cultivated under controlled conditions, ensuring high yield and purity while minimizing environmental impact. Despite their potential, challenges remain in optimizing extraction techniques, enhancing structural characterization, and scaling up production for commercial applications. This review provides an overview of the principal biological activities of PSs from eukaryotic microalgae and their possible use as ingredients for cosmetic applications. Challenges to address to implement their use as products to improve human health and wellbeing are also discussed.

## 1. Introduction

Polysaccharides (PSs), the most abundant form of carbohydrates present in nature [1], perform several vital functions, including cell–cell communication, cell adhesion, protection, structural role, stimuli responsiveness, blood clotting and relevant roles in immune system regulation [2,3]. These natural macromolecular polymers are composed of monosaccharide units linked together by glycosidic bonds, forming linear or branched chains. In addition to their carbohydrate backbone, PSs can be covalently bound to peptides, amino acids and lipids, further diversifying their chemical properties and biological functions.

The classification of PSs is based on their monosaccharide composition, chain length, branching patterns, and electrolytic nature [1,4], which in turn influence their physical, chemical, and biological properties. Structural modifications such as sulfation, phosphorylation and carboxymethylation can render PSs more stable, non-toxic and biodegradable, making them suitably good candidates for applications in industries such as pharmaceuticals, food, energy, materials science, and environmental base solutions [1,5].

PSs are widely distributed in nature and can be found in terrestrial and marine organisms including echinoderms, ascidians, crustaceans, seaweeds, microalgae, cyanobacteria and marine bacteria. Several marine organisms have been screened for the presence of polysaccharides that are potentially useful for their properties. Some examples include sulfated fucoidans, hyaluronic acid, chondroitin sulphate, dermatan sulphate and heparin sulphate isolated from sea urchins and sea cucumbers [6,7,8,9], and chitin, the most abundant polysaccharide present in nature after cellulose [10], obtained from crustaceans’ exoskeletons [11,12]. Seaweeds are among the richest source of several different PSs. Well-known examples are agar and carrageenans, galactans typical of red macroalgae, which are widely used as thickening and gelling agents [13], although they also showed immunostimulant, anticoagulant, antiviral and antitumor activity [5,14,15,16], suggesting other possible applications. PS content in macroalgae may be considerably high, such as for the green seaweed *Ulva* sp. in which it ranges from 29 to 51% of the obtained extracts [17], mainly characterized by the presence of ulvans, interesting for their bioactive and rheological properties.

**Figure 1:**
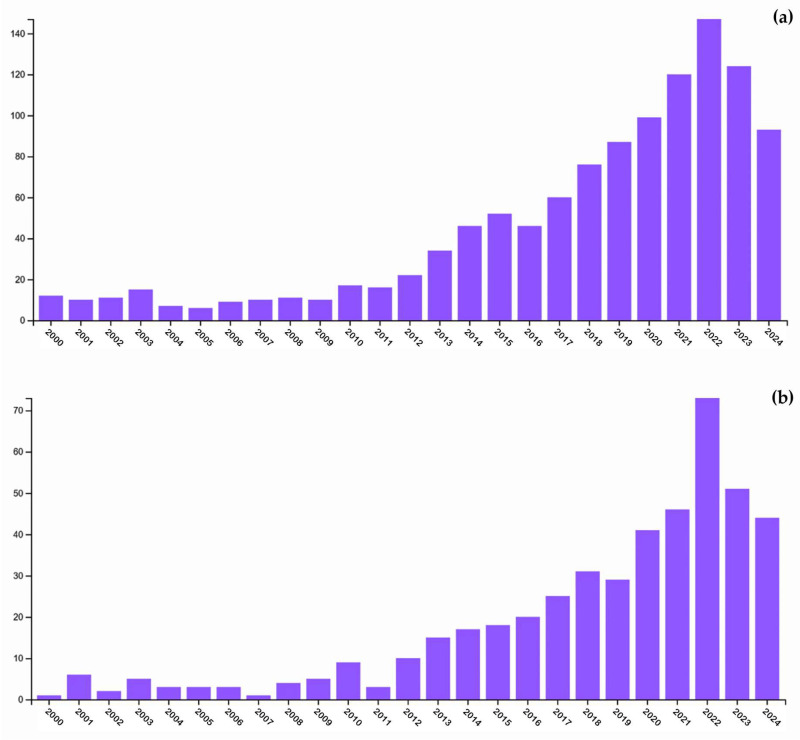
(**a**) Trend of papers focusing on polysaccharides from microalgae. Data were retrieved from Web of Science using the query microalg* polysaccharide* (topic) from 2020 to 2024. (**b**) Trend of papers focusing on bioactive polysaccharides from microalgae. Data were retrieved from Web of Science using the query microalg* polysaccharide* (topic) and Activ* (search within topic).

While terrestrial plants and macroalgae have historically been the primary sources of PSs for commercial and industrial use, microalgae are emerging as a promising alternative due to their high growth rates, adaptability to controlled cultivation, and ability to synthesize structurally diverse PSs with bioactive properties. Indeed, besides their biological functions and ecological roles, microalgae PSs are increasingly recognized for their potential in diverse industrial applications, as they are more safe, stable and biocompatible than other PSs [18]. Compared to macroalgae and terrestrial plants, microalgae offer advantages in large-scale cultivation, as they can be grown rapidly under controlled conditions without the limitations of seasonal variation or arable land use. Furthermore, microalgae can be cultivated in closed photobioreactors or open pond systems, allowing for sustainable and scalable PS production. These features, combined with the increasing global interest in bioactive natural products, position microalgal PSs as a promising resource for next-generation biotechnological innovations for improving human health and wellbeing. The growing interest in microalgae-derived PSs and their bioactivity is evident from the substantial rise in publications on this topic in recent years (Figure 1), with the highest value in 2022.

In this review, we provide an overview of key features and biological activities of PSs derived from eukaryotic microalgae and explore the challenges associated with their exploitation for human health and wellbeing. By consolidating current knowledge, we aim to contribute to the growing interest in microalgal PSs as valuable bioresources for sustainable innovation.

## 2. Microalgal Polysaccharides: Diversity and Structure

**Figure 2:**
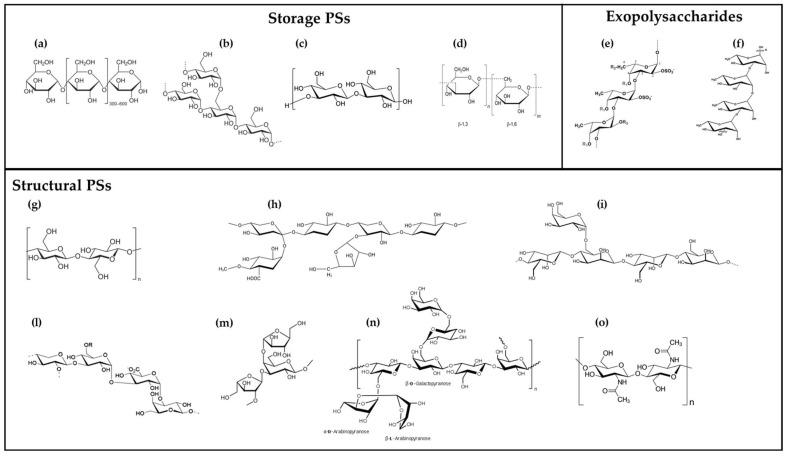
Some examples of the main polysaccharides detected in microalgae. (**a**) amylose; (**b**) floridean starch (semi-amylopectin); (**c**) paramylon; (**d**) chrysolaminarin; (**e**) generic structure of sulfated fucans [47]; (**f**) backbone repeating unit sequence →2)-α-L-Rha (1→3)-α-L-Rha(1→ of *Chlorella vulgaris* EPS [18]; (**g**) cellulose; (**h**) hemicelluloses; (**i**) example of mannan: (**l**) acidic heteropolysaccharide [(2 or 4)-ß-d-Xylp-(1→3)]m-α-d-Glcp-(1→3)-α-d-GlcpA-(1→3)-l-Galp-(1→] from the red microalga *Porphyridium* sp. [48]; (**m**) repeating unit →2)-α-l-Araf-(1→3)-[α-l-Araf-(1→4)]-β-d-Galp-(1→ of arabinogalactan from *Chlorella pyrenoidosa* [49]; (**n**) example of arabinogalactan; (**o**) repeating unit of chitin.

Eukaryotic microalgae produce PSs principally composed of monomers of xylose, galactose, glucose, rhamnose, mannose, fucose and fructose [19,20,21,22]. They are synthesized in the chloroplast during the Calvin Cycle and sulfation occurs in the Golgi apparatus [20,22]. Microalgal PSs can be classified into three major categories based on their function: storage PSs, structural PSs, and exopolysaccharides (EPSs) [23] (see Figure 2 for some examples of the main polysaccharides detected in microalgae).

Storage PSs serve as energy reserves and differ among microalgal taxa: starch is the primary storage PS in Chlorophyta, alongside floridean starch in Rhodophyta, chrysolaminarin in Haptophyta and Heterokontophyta, and paramylon in Euglenophyta and Chlorarachniophyta. These PSs are composed of glucose residues linked by α- or β-glycosidic bonds, with varying degrees of branching and solubility. Except for chrysolaminarin that is stored in vacuoles, the other storage PSs are accumulated as granules in chloroplasts for starch and in the cytosol for the others [24,25].

Structural PSs are primarily located in the cell wall and contribute to mechanical strength, protection, and environmental resilience. In microalgae, cell-wall PSs are mainly composed of cellulose and hemicelluloses, with additional sulfated PSs present in certain species [20], such as for the sulfated glucuronomannan isolated from the cell wall of the diatom *Phaeodactylum tricornutum* [26]. The composition of cell-wall PSs varies across different microalgal groups: the Chlorophyta cell wall is mainly composed of monomers of glucose, rhamnose, glucosamine, galactose and mannose. The cell wall of this phylum can be characterized by the presence of different molecules: (i) 2-keto-sugars and mannans (as in Chlorodendrophyceae like *Tetraselmis* and *Scherffelia* spp.), (ii) β-(1,3) and β-(1-4) glucan, associated with algaenan biopolymers [27], and (iii) mannans and arabinogalattans (as in Trebouxiopyceae like *Botryococcus* spp., in Chlorellaceae, and also typical for *Clamydomonas* spp. and *Hematococcus* spp.). Interesting is the presence in some Chlorellaceae of a rigid cell wall constituted by glucosamine and chitin-like PSs [28,29]. The Haptophyta and Eustigmatophyta cell wall consists principally of monomers of glucose, like β-D-glucan in *Isochrysis galbana* or in *Nannochloropsis oculate* [30,31]. In diatoms (Bacillariophyta), the cell wall consists of three parts: an organic layer attached to the cellular membrane, a siliceous layer, and the outermost layer made of extracellular polymeric substances. PSs in diatoms are predominantly composed of galactose, mannose and glucosamine monosaccharides; some species also present chitin as the cell wall fibrillar PS, and genes for chitin synthesis and deacetylation are present in some species like *P. tricornutum* and *Thalassiosira pseudonana* [32]. The presence of sulfated PSs has been detected in *P. tricornutum* as sulfated glucuromannan and as sulfated fucans EPS in *Thalassiosira weissflogii* and *Chaetoceros socialis* [26,33,34]. In the Rhodophyta cell wall, the rigid part is absent, presenting only a polysaccharidic layer of variable thickness mainly constituted by galactose, glucose, xylose, and glucuronic acid [25,35,36]. In many species like *Porphyridium cruentum*, *P. purpureum* or *Rhodosorus* spp., the presence of sulfated hetero-PSs is also reported [37,38,39].

EPSs are another important class of microalgal PSs and can be either closely associated with the cell wall or released into the surrounding environment. These complex heteropolymers, often rich in sulphate groups and uronic acids [40,41], play essential ecological roles such as cell adhesion, biofilm formation, water storage, protection against environmental stressors, and interaction with microorganisms [24,25]. The chemical composition of EPSs is highly variable, even among species within the same genus, making them a subject of growing interest for industrial applications. In benthic diatoms, EPSs facilitate gliding motility and surface attachment, while in planktonic microalgae, they contribute to buoyancy regulation and defence mechanisms [41,42,43]. In Rhodophyta and Chlorophyta, EPSs are composed of hetero-polymers of galactose, xylose and glucuronic acid [44,45]; instead, in diatoms, in the form of mucilage, they are essentially composed of rhamnose [21,46].

As the chemical structure is very complex and difficult to be characterized, even with glycochemistry methods, only few EPSs have been fully characterized so far, and available data show that there is a great variety in EPS chemical structure among microalgae belonging to the same genus [24]. The structural diversity of polysaccharides from microalgae has been described in detail by Prybylski and co-authors [50], who reported the % of sugar monomers and the main structural features for diverse groups of microalgae. This diversity confers different biological properties to the EPSs that can be of interest for possible applications in the cosmetic, nutraceutical and pharmaceutical fields.

## 3. Factors Influencing Polysaccharide Production in Microalgae

The biosynthesis and accumulation of PSs in microalgae is influenced by abiotic factors such as temperature, salinity, light, pH, nutrient uptake, and culture growth phase [20,51]. The most important factor influencing PS production is light, in particular light radiation, illumination intensity and its spectral composition [22,52,53]. For example, an increase in photosynthetic photon flux density stimulates the production of PSs in *P. purpureum*. As for spectral composition, blue and red spectra are particularly efficient at increasing PS production in the same microalgae [54], but also yellow and green wavelengths may have similar effects [53,55].

Nutrient availability, particularly nitrogen, phosphorus, and sulphur, is another critical factor affecting PS accumulation [56]. Nitrogen is important for photosynthetic systems and microalgae metabolism; phosphorus is fundamental for the synthesis of DNA or ATP, and sulphur is vital for protein synthesis. Consequently, their limited uptake triggers the accumulation of reserve compounds, a phenomenon similarly observed under increased salinity or temperature changes [20,22]. For example, the reduction in nitrogen concentrations in *Dunaliella tertiolecta*, *Dunaliella salina*, *Chlorella minutissima*, and *Desmodesmus* sp. cultures increases polysaccharide production by 1.5 times [20,57]. Regarding salinity, to maintain homeostasis and protect the cell integrity, microalgae produce more PSs [20,22,53]. Indeed, a study reported that the marine microalgae *Schizochytrium* sp. increase chitin and cellulose production as an adaptation to low-salinity environments (6 g/L) [58,59]. However, hyper-salinity also enhances PS production in microalgae, as observed in *D. salina* when exposed to NaCl concentrations ranging from 0.5 to 5 M [22,60]. Temperature changes could, in combination with light energy, affect nutrients’ uptake and membrane structures, which results in an overproduction of PSs [22,53,61]. For example, a higher production of PS was obtained from *Chlorella vulgaris* at a temperature of 25 °C, but under the same conditions, a lower PS content was extracted from *Phaeodactylum tricornutum* and *Nannochloropsis* sp., highlighting that this factor is species-dependent [20,62].

Other studies reported that pH values could influence microalgal growth, cell metabolism, nutrient availability, ammonia toxicity and PS production [20]. Authors observed that low pH or a combination of determinate value of pH and nutrients increases PS accumulation [63]. Finally, aeration is another important factor for microalgae growth, and it seems to influence PS production [22], although it is still not clear if its effect is directly linked to PS production, better light penetration, or better CO_2_ uptake. In general, modification of optimal growth conditions and the consequent stress causes an increase in PS production with species-specific characteristics [22,51].

**Figure 3:**
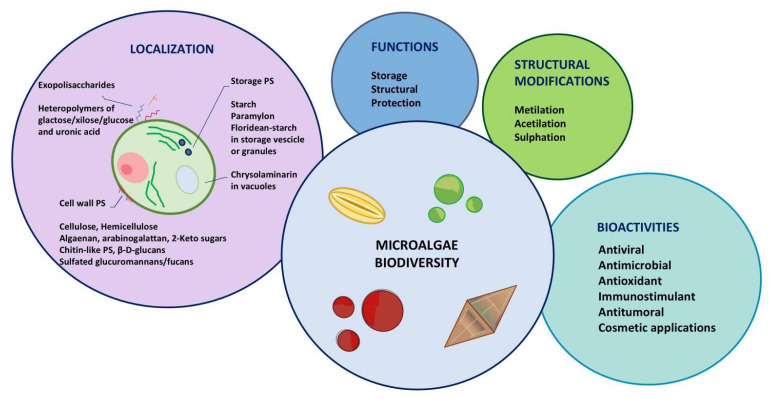
Synoptic representation of possible localization, functions, structural modifications and bioactivities of microalgal polysaccharides.

Figure 3 summarizes the key features of microalgae polysaccharides and their main biological activities that are described in the following sections.

## 4. Biological Activities of Microalgal Polysaccharides

PSs possess different biological activities [47,64,65] that are useful for improving human health, including antiviral, antimicrobial, immunostimulant, antioxidant, and antitumor activities. In addition, they can also be used for cosmetic applications and for the synthesis of compounds for applications in the biomedical field, such as for iduronic acid that is used as a precursor in the synthesis of heparin analogues [22,66,67].

### 4.1. Antiviral Activity

Currently, several emerging viruses are rapidly isolated, many of which are drug- or vaccine-resistant; therefore, there is a great need to find new antiviral agents [64,68,69]. Microalgae PSs could be used for this aim, thanks to their chemical structure and electrolytic nature, particularly due to the presence of negative electric charges that are able to interact with positive charges present on the virus envelope or on the bacterial cell surface [68,70,71,72]. Many authors observed that these molecules can act differently on viruses [73], for example, by inhibiting the penetration into host cells, the synthesis of viral proteins inside the cells [73], the activity of enzymes involved in the viral life cycle (thus affecting replication), or by inducing interferon production [74]. This variability in the effect depends on the size and degree of sulfation, the composition of the sugar units, the molecular weight, and the diversity of the linkage chemistry [68,72,73,75]. A list of microalgae species producing bioactive polysaccharides with antiviral activity, including their monomeric composition (when available), is reported in Table 1. Some authors showed that sulfated PSs, composed of xylose, glucose and galactose, extracted from red microalgae, *Porphyridium* sp., *P. aerugineum* and *Rhodella reticulata*, inhibit the infection of Vero cells by *Herpes simplex* viruses 1 and 2 and Varicella zoster virus. In particular, the most efficient effects were shown by PSs extracted from *Porphyridium* spp., linked to their highest degree of sulfation. These PSs act on viruses by inhibiting the cytopathic effect on cell cultures, blocking life cycle steps of viruses, or preventing replication and reinfections within host cells [74]. The same SP exerts in vivo antiviral activity towards HSV-1 in murine and rabbit models, preventing the development and the symptoms of the infection at 100 µg/mL [76]. PSs extracted from the same species also showed antiviral effects against Moloney murine sarcoma virus and Moloney murine leukemia virus, reducing virus replication and malignant cell transformation, particularly at early stages of infection, probably acting on virus penetration into host cells [77]. In addition, Lee and co-workers observed that a PS composed of fucose, xylose, galactose, mannose and rhamnose isolated from *Navicula directa* acts against enveloped viruses such as *Herpes simplex* virus 1 and 2 and influenza A virus by inhibiting penetration inside the cells [78]. Other authors studied the potential antiviral effects exerted by a sulfated EPS (p-KG03) extracted from the marine dinoflagellate *Gyrodinium impudicum* against Influenza virus type A [68]. They demonstrated that this EPS primarily reduces influenza virus type A infection in Mardin–Darby canine kidney cells; in particular, this compound seems to have double action, both inhibiting virus binding to the host cell and preventing virus internalization before the early stages of the virus replication cycle. Thus, this homo-PS, composed of galactose, could be used as a possible antiviral drug against influenza viruses, thanks also to the high growth rate of the microalgae in controlled conditions and the thermodynamic stability of the molecule, which allows it to be stored for a long time in stock preparations. The dinoflagellate *Cochlodinium polykrikoides* also produces two sulphate PSs (A1 and A2) with antiviral activity against viruses of the Paramyxoviridae family, such as HIV1, HSV, RSVA-long and parainfluenza (MLSV) [72,79]. Despite extensive research, challenges remain in using PSs as antiviral drugs. These include their high molecular weights that prevent cell membrane penetration and their structural complexity that hinders enzymatic degradation, leading to persistence and potential cytotoxicity and, in some cases, a limited understanding of their mechanisms of action. For these reasons, it would be advisable to study these molecules and their action more deeply [74].

### 4.2. Antimicrobial Activity

Bacteria can be dangerous for humans because they cause the most community-acquired and nosocomial infections. Furthermore, they are capable of resisting the effects of antibiotics, with a particular increase in incidence in recent years, also due to an uncontrolled use of antibacterial drugs with negative impacts on human health [84,85]. Consequently, it is necessary to enhance and deepen this research for a better understanding of antibacterial resistance mechanisms, preventing the spread of resistance and discovering new, more efficient natural antibacterial agents, with less side effects and toxicity. In this context, microalgae PSs could be an important resource to develop new drugs, especially against drug-resistant bacteria such as *Pseudomonas aeruginosa*, *Staphylococcus aureus*, *Helicobacter pylori* and *Salmonellae* spp. [71,86]. Indeed, many authors found antibacterial activities against *Staphylococcus aureus* in PSs extracted from *Rhodella reticulata*, *Porphyridium cruentum*, *Isochrysis galbana* and *Nannochloropsis* sp. [47,87,88,89]. The last two also possess the same activities against *Pseudomonas aeruginosa, Escherichia coli* and three species belonging to the *Candida* genus [87]. Microalgae PSs exert antibacterial activity thanks to the link between cell-surface PS glycoprotein receptors and bacterial components like the cell wall, cytoplasmic membrane and DNA. This bond prevents the adhesion of bacteria; thus, microalgae PSs seem to have anti-adhesive properties [52,90]. In particular, several studies showed that this feature mainly belongs to sulfated PSs due to their chemical structure [52,53,70]. For example, sulfated PSs extracted from *Tetraselmis* sp., *Neochloris oleobundan* and *P. tricornutum* showed anti-adhesive activities against *Helicobacter pylori* [91]. In addition, a sulfated galactan obtained from *P. cruentum* inhibits *Salmonella enteritidis*, *Staphylococcus aureus* and *E. coli* growth [88] and a hydrogel based on *Porphyridium* spp. sulfated PSs was shown to have potential to form a barrier against bacteria [92]. Nevertheless, non-sulfated polysaccharides also show antibacterial activity. One example is the finding by Najdenski et al., who screened nine cyanobacterial and ten microalgal strains against eight significant food-borne pathogens [89] and found interesting activity of an EPS isolated from the Rhodophyta *Rhodella reticulate*. The EPS inhibited the growth of *S. aureus* and *Bacillus cereus* with an MIC of 0.25 mg/mL and *Streptococcus pyogenes* with an MIC of 1 mg/mL. An in vivo study demonstrated that a polysaccharide extract from *Chlorella pyrenoidosa* exhibited antibacterial activity against *Listeria monocytogenes*. In particular, acting as immunomodulators, two different doses of the Chlorella extract significantly reduced the number of bacterial colony-forming units (CFUs) in spleen cell cultures from BALB/c mice three days after Listeria infection: 43,310 CFUs with 0.1 mg and 5317 CFUs with 4 mg compared to 92,200 CFUs in the control [93]. In addition, microalgae PSs could act against bacteria indirectly, through their use in nanobiotechnology, in particular in the production of nanoparticles. Indeed, their presence in these particles seems to influence drug cell penetration and antimicrobial effects. One example is represented by silver nanoparticles synthesized using *Botryococcus braunii* and *C. pyrenoidosa* EPS, which serve as both reducing and stabilizing agents. The study demonstrated the antibacterial activities of these nanoparticles against drug-resistant bacteria *Staphylococcus aureus* and *Escherichia coli*, showing that the polysaccharide-capped silver nanoparticles may have great potential in wound healing and tissue engineering, as well for drug delivery [94]. The combination of gold and zinc nanoparticles with microalgae PSs also enhances antimicrobial activity. Specifically, gold nanoparticles stabilized with *Nitzschia* sp. PSs improved drug molecule penetration into bacterial cells, showing greater efficacy against *E. coli*, *P. aeruginosa*, and *S. aureus* [95]. Similarly, the combination of zinc nanoparticles with chitosan and a hydrogel derived from *Porphyridium* sp. PSs further improved antimicrobial properties [53].

Not only bacteria threaten human health, but also fungi that also affect animals and plants. Microalgae PSs could represent a source of new antifungal agents to fight high fungi toxicity and their drug resistance. For example, PSs extracted from *Tetraselmis* spp. have antifungal activities against *Candida albicans* and *Penicillium italic*, which affect humans and plants, respectively [96]. Another example is a study by Kravolec et al., who demonstrated the antifungal activity of polysaccharides extracted from *C. pyrenoidosa* against *Candida albicans*, as evidenced by a decrease in CFUs in kidney cell cultures from BALB/c mice 12 days after *Candida* infection [93]. In addition, an EPS extracted from *Microchloropsis gaditana* showed high antifungal activity (84.8% growth inhibition) against *Cladosporium cladosporioides* at a concentration of 500 mgGlcEq/L [97]. Some fungi are considered emerging pathogens due to their zoonotic potential, such as those belonging to the Microsporidia phylum. In this contest, researchers found anti-microsporidian activities in PSs extracted from *Porphyridium marinum*, in particular against *Nosema ceranae* that affects honeybees, insects fundamental to the ecosystem [78]. A list of microalgae species producing polysaccharides with antimicrobial activity, including their monomeric composition (when available), is reported in Table 2. Despite the potential of microalgae PSs as antimicrobial agents, they have not been developed yet to reach the market.

### 4.3. Antioxidant Activity

Oxygen free radicals and non-radical derivatives of oxygen, like superoxide anions, hydrogen peroxide and hydroxyl radicals, are naturally produced consequently to the metabolic activity and are fundamental in some biological functions, such as intracellular signalling in cell differentiation and apoptosis [4,100,101]. However, an excess of their production could cause oxidative stress with toxic effects due to the structural modifications of fundamental biomolecules like DNA, proteins and lipids [1,100]. This could cause several degenerative, metabolic and other diseases such as tumours, diabetes, cardiovascular pathologies, immune system damage and also ageing [1,22,100,101]. The human body has some defences to contrast oxidative stress that consist of enzymatic and non-enzymatic mechanisms, but they are often insufficient, so it is necessary to introduce exogenous antioxidants [101]. Natural products, such as microalgae PSs, are gaining significant attention for their antioxidant potential, in particular from the pharmaceutical and food industries [52,102,103]. Antioxidant activities of microalgae PSs are influenced by numerous factors that depend on the microalgae culture growth conditions, microalgae species (even within the same genus), the extraction method and, overall, their chemical structure [101,102]. In particular, regarding the latter, it seems that the molecular weight of PSs plays an important role in increasing antioxidant activity. Indeed, PSs obtained from high-molecular-weight carbohydrates have higher scavenging activities compared to the precursor, but at very low molecular weight the biological activity decreases; thus, it is of great importance to identify the right range of molecular weight of certain bioactive polysaccharides [101]. The monosaccharide composition is also relevant, especially the number of the same sugar unit and the presence of galactose in the macromolecule [104,105].

As for other biological activities of microalgae PSs, antioxidant properties are influenced by sulfation degree and by sulphate group position, due to the anionic nature of this chemical group [101,103]. There are many examples of microalgae SPs with antioxidant activity in the literature [106]. A list of microalgae species producing bioactive polysaccharides with antioxidant activity and their monomeric composition (when available) is reported in Table 3. For example, it has been observed that *Porphyridium* sp. PSs prevent linoleic acid autoxidation and provide protection to NIH/ 3T3 cells against oxidative damage caused by FeSO_4_ [106]. Green microalgae PSs also have antioxidant activities, like *Chlorella vulgaris* and *Isocrysis galbana*, and PSs stimulate the activity of antioxidant enzymes such as catalase and superoxide dismutase [72,107]. Diatom PSs possess scavenging activities against free radicals, superoxide anions and nitric oxide, such as those from *Navicula incerta* [104,108], or against hydroxyl radicals, such as chrysolaminarin extracted from *Odontella aurita* [21,109]. Furthermore, some studies suggest that the presence of other molecules enhances PSs’ antioxidant potential, such as in the presence of phenols, flavonoids, and proteins. Thus, the purification procedure is an important aspect to keep into consideration when evaluating antioxidant activity.

Microalgae PSs could also provide indirect protection against oxidative stress by influencing gut microbiota composition or by activating gastrointestinal immune cells, but the mechanism underlying this effect is still unclear [101].

Only few studies investigated the antioxidant activities of microalgae polysaccharides in vivo, and the literature remains limited regarding the molecular mechanisms underlying the action of these compounds. However, Yi et al. (2021) demonstrated the antioxidant potential of polysaccharide extract from an Antarctic ice alga, *Chlamydomonas* sp., in D-galactose-induced ICR mice [110] in vivo. In this study, PSs from the Antarctic microalgae significantly improved thymus, brain, heart, liver, spleen, and kidney index values in mice subjected to oxidative damage induced by D-galactose. Furthermore, PS treatment reduces aspartate aminotransferase, alanine aminotransferase and alkaline phosphatase levels in mouse serum. These enzymes are markers indicative of liver function; indeed, their high level in the serum is linked to liver tissue damage, caused by oxidative damage. Additionally, the treatment normalized the levels of several oxidative stress markers, including the activity of superoxide dismutase (SOD), catalase (CAT) and glutathione peroxidase, as well as glutathione and malondialdehyde content, suggesting an action against oxidative stress damage. Regarding the molecular mechanism, this study suggests that *Chlamydomonas* sp. PSs up-regulate the expression of key antioxidant and stress-related proteins like neuronal nitric oxide synthase (nNOS), which protects against oxidative damage to different tissues such myocardium and muscle; endothelial nitric oxide synthase, which plays a role in counteracting oxidative stress-induced vascular ageing; and cuprozinc-superoxide dismutase, manganese superoxide dismutase, and catalase, the main antioxidant enzymes. Additionally, they up-regulate the expression of heme oxygenase-1 (HO-1), a stress-responsive enzyme involved in heme metabolism with known anti-inflammatory and antioxidant functions, and nuclear factor erythroid 2-related factor 2 (Nrf2), a transcription factor that regulates antioxidant defence genes. Its activation can promote the expression of HO-1, CAT, SOD, and γ-glutamylcysteine synthetase (γ-GCS), the enzyme responsible for glutathione synthesis. In the presence of oxidative stress, Nrf2 activates the expression of the antioxidant enzyme NAD(P)H dehydrogenase [quinone] 1 (NQO1). Overall, these findings highlight the promising antioxidant and cytoprotective properties of polysaccharides from Antarctic *Chlamydomonas* sp., along with initial insights into the molecular pathways involved, notably through the Nrf2/HO-1 axis and NO synthase signalling.

Similar results were obtained for heteropolysaccharides purified from *Golenkinia* sp. and *Chlorella sorokiniana* [105]. These carbohydrates, composed mainly of galactose, had antioxidant properties in vitro, decreased MDA concentration and increased SOD and T-AOC content in HepG2 cells treated with H_2_O_2_, suggesting that these PSs can diminish the damage caused by free radicals stimulating the antioxidant enzyme system of the cells. Moreover, these PSs promoted the dissociation of the Keap1–Nrf2 complex by down-regulating the Keap1 gene and up-regulating the Nrf2 gene, ultimately activating the expression of the antioxidant enzymes NQO1 and HO-1. These results confirm that these microalgae PSs exert their antioxidant activity through the Nrf2/HO-1 signalling pathway.

The antioxidant effect of a PS isolated from *C. pyrenoidosa* was tested on *Caenorhabditis elegans* as an in vivo animal model system. The study revealed an increase in the level of SOD and the reduction in MDA and ROS content in animals exposed to the PS, which proved its antioxidant capacity [111]. Moreover, the individual exposed to the PS showed an up-regulation of ageing-related genes Daf-16 and Skn-1 and extended longevity. These results were corroborated by evidence of changes in the expression levels of miR-48 and miR-51, which directly influence the levels of Daf-16 and Skn-1.

Considering the current status of the knowledge in this field, future studies must consider the occurrence and combination of different factors to clarify the variability of antioxidant activity shown by PSs and find the most appropriate method to obtain PSs with higher and stable antioxidant activities.

### 4.4. Immunomodulatory Activity

The immune system performs the fundamental functions of defence of the organism and prevention from diseases, inflammation and tumours. Microalgae PSs may contribute to improving the defence system and preventing bacterial and viral infections, tumour progression and other chronic diseases through the stimulation of both the innate immune and adaptive responses [4,21,29,52,70,127]. In the literature, there are several studies about microalgae PSs with immunostimulant activity, in particular EPS and SPs. For example, the red microalgae *P. cruentum* EPSs were found to have immunomodulant activity, influencing nitric oxide (NO) production in macrophages and enhancing the production of splenocytes [128]. An in vivo study on the effect of an SP obtained from the same microalga showed the capability to modify the white blood cell differentiation and peritoneal macrophage profiles in mice [39]. A sulfated EPS extracted from *G. impudicum* was shown to increase immunological activities of natural killer (NK) cells and macrophages in mice administered with a single dose of p-KG03 (100 or 200 mg/kg of body weight). The EPS also stimulated the production of antibodies and cytokines IL-1β, IL-6, and tumour necrosis factor-alpha (TNF-α), which are involved in pro-inflammatory and anti-inflammatory processes [129,130]. The authors suggested that this immunostimulatory effect may improve the tumoricidal activities of macrophages and NK cells in vivo. A sulfated hetero-PS extracted from *Tribonema* sp. showed similar effects as it improved macrophage cell viability and induced the release of cytokines IL-6, IL-10 and TNF-α [131]. In addition, SPs extracted from *C. stigmatophora* and *P. tricornutum* showed anti-inflammatory and immunomodulatory activities in vivo and in vitro. Specifically, the *C. stigmatophora* PS extract showed immunosuppressant effects, while the *P. tricornutum* extract displayed immunostimulatory effects [132]. Another example of a PS with immunostimulant activity in vivo is the chrysolaminarin-enriched extract from *P. tricornutum*. This PS enhanced the expression of the pro-inflammatory cytokine IL-1β in the kidney, spleen, and intestine of the fish *Senegalese sole*. The activation of this cytokine serves as a mediator of chrysolaminarin’s effects, promoting the production of cytokines and the activation of macrophage migration and phagocytosis [133]. In vivo experiments conducted on a PS derived from *C. pyrenoidosa* demonstrated that its oral administration to male mice (1 and 2 g/kg of body weight) significantly enhanced phagocytosis [134]. A study by Wu and co-authors [135] provided preliminary insights into the potential mechanism of a sulfated heteropolysaccharide from *Chlorella sp*. in regulating the immune response of RAW264.7 macrophages. This SP induced the production of NO, TNF-α, and IL-6 in a dose-dependent manner, with peak activity observed at 6 µg/mL. Transcriptomic analysis of RAW264.7 macrophages following SP treatment revealed significant up-regulation of immune-related genes, including NFKB1, IL-6, and IL-1β. Furthermore, differentially expressed genes were significantly enriched in immune-related biological processes, such as the Toll-like receptor (TLR) signalling pathway, cytosolic DNA-sensing pathway, and C-type lectin receptor signalling pathway [21,70,127].

Table 4 provides a list of microalgae species producing immunostimulant polysaccharides and their monomeric composition (when available).

Overall, these data suggest that the primary mechanism through which PS exert their effects is by binding to receptors on immune cells—such as macrophages, dendritic cells, monocytes, NK cells, and lymphocytes—leading to their activation. This activation induces the release of cell-mediating molecules, including cytokines, TNF-α, interferon-gamma (IFN-γ), NO, and immunoglobulins [21,70,127]. These effects result in a general stimulation of the immune response, enhancing defence mechanisms against infection while simultaneously down-regulating inflammation in in vivo studies, which encourages the exploitation of microalgae PS for application as immunostimulants.

### 4.5. Antitumor Activity

The global effort to find novel and effective compounds to fight cancer disease is a critical need of our time. Indeed, cancer is a disease that affects millions of people worldwide, and its causes are complex and multifactorial [142]. The incidence of cancer is one of the major causes of illness and death on a global scale, with about 19.9 million new cases and 9.7 million deaths registered for 2022 [143]. The progression of cancer can be attributed to various factors including damage to DNA, anomalies in DNA repair mechanisms, weakened tumour suppression activity, and the stimulation of angiogenesis and metastasis processes, all of which contribute to the survival and growth of cancer. With a prediction of a global increase of 63.4% in cancer cases by 2045, it is imperative that science and medicine work collaboratively to discover new therapies and solutions to contrast this pressing issue [143]. Survival rates for cancer disease have improved in recent years, but continued research and development of therapies is crucial for the future.

Compounds from natural sources offer new therapeutic strategies to combine with classical approaches such as chemotherapy and radiotherapy that can cause many adverse reactions in patients. The interest in this field is very high and many are the examples of microalgae-derived products that can produce antitumor bioactive compounds [144,145]. Microalgae PSs in many cases demonstrate cancer-preventive and antitumor activity, also considering the derivatives and analogues obtained by chemical modifications of the natural compound. These molecules could have a direct effect on cancer cells or influence tumour development at different stages of carcinogenesis, suggesting their interesting potential as alternative or synergic treatment drugs [146]. For example, EPSs from the Rhodophyta *Porphyridium* sp. have displayed different anticancer activities. Geresh et al. analyzed the action of EPS with high sulphate content towards T-cell lymphoma line 24-1, reporting an inhibition of cancer cell proliferation of 80% [147]. Another study also assessed the antitumor activity of a sulfated EPS named PcrPSH tested in in vitro and in vivo hamster models against Graffi myeloid tumours. This treatment produced a consistent decrease in mortality rate. The activity of this compound is associated with an activation of apoptotic pathways, but evidence also shows an increase in phagocytic ability of macrophages, suggesting also an immunostimulant action [148]. Recently, a sulfated hetero-PS purified from the microalga *Tribonema* sp. showed immunostimulatory activity, enhancing macrophage cell viability, and dose-dependent anticancer activity towards HepG2 cells; this PS induces apoptosis in cancer cells, affecting mitosis and cell cycle progression [131]. Another report on PS anticancer activity regards the SP identified as PPS0 and produced by *Pavlova viridis* (Chrysophyta), which is mainly constituted by fructose, glucose, mannose and uronic acid. This is also an example of how the reduction in molecular weight could improve biological activity. Often PSs with large molecular weight show poor water solubility, a property that limits the research on biological activities [149]. For this reason, PPS0 was degraded with H_2_O_2_–vitamin C, assisted by ultrasonic waves, which led to the formation of two products, PPS1 and PPS2, that showed immunoenhancing and anticancer activity, respectively. In particular, the anticancer activity was assessed in vivo in transplanted S180 mice (sarcoma model) showing an inhibition of tumour growth of 56% compared to the control [150]. Also, *Sarcinochrysis marina* sulfated polysaccharides exhibit antitumour activity against Sarcoma 180, significantly inhibiting tumour growth at a concentration of 200 mg/kg/day in an in vivo model of female Kunming mice. Moreover, treatment with these polysaccharides led to an increase in spleen and thymus indices, as well as enhanced lymphocyte proliferation. These findings suggest that the observed antitumor effects may be attributed, at least in part, to their immunostimulatory properties [151]. Previously, the same authors observed similar antitumor effects in mice implanted with Sarcoma 180 and treated with S-EPS extracted from *Porphyridium cruentum* [128]. Another study evaluated the anticancer activity of PCEPS and EPS obtained from *Parachlorella kessleri* extract, actively inhibiting colon carcinoma growth in both HT-29 and CaCo-2 human cells. This compound is also active in vivo in murine colon carcinoma peritoneal dissemination model CT26, where there is evidence of the stimulation of immune response [152]. Interesting anticancer activity has been found from the freshwater diatom *Nitzschia palea*, producing an ESP that is active in vitro towards A549 adenocarcinoma lung cancer cells with cellular death mediated via apoptosis [153]. Active PSs purified from the diatom *P. tricornutum* showed various bioactivity; in particular, the antiproliferative activity of this compound was assessed towards HepG2 cells, revealing an interference in cell cycle and mitosis and showing, at the highest concentration of the compound, an inhibition of cellular growth of 60% compared to the control [149]. The compound was shown to be mainly constituted by xylose, fucose, glucose and a high content of glucuronic acid. Analyzing marine dinoflagellates, the toxic *Gymnodinium* sp. produced an EPS named GAP3P; this molecule presents potential anticancer activity towards leukemia K562 cells, showing catalytic inhibition of DNA Topoisomerase I and II. Indeed, GAP3P has also been found active in vitro against other cancer cell lines [154]. A list of microalgae species producing potential antitumor polysaccharides is reported in Table 5.

### 4.6. Applications for Cosmesis

The use of marine metabolites in cosmesis is nowadays increasing, driven by the wide variety of biologically active compounds and the growing consumer awareness and preference for eco-friendly, natural ingredients [160]. PSs can be readily applied in the cosmetic industry as gelling, moisturizing or thickening agents. Their use as active ingredients in personal care products aims to enhance skin structure and appearance. Of particular interest are applications for blemish prevention and skin damage repair, seborrhoea regulation, and inflammation reduction [161]. Some bioactive molecules derived from microalgae can be incorporated into cosmetic formulations due to their beneficial properties, such as accelerating the wound healing process, acting as a UV filter and reducing signs of ageing, including wrinkle formation and skin hyperpigmentation [160,162,163]. Among these, EPSs and glucosyl glycerols are the most extensively studied molecules, primarily recognized for their role in fighting oxidative stress and skin ageing [164].

Notably, Rhodophyta and, in particular, the genus *Porphyridium* have shown the presence of a sulfated EPS with acidic characteristics, which holds significant potential for cosmetic applications. This molecule acts as a hyaluronidase inhibitor and possesses anti-inflammatory and anti-allergic properties [42]. Another study identified ten different sulfated PSs from *P. cruentum* showing a 96% inhibition of hyaluronidase activity at the tested concentration ranging from 0.25 to 2.5 mg/mL, as well as 46% inhibition of elastase enzymatic activity at a concentration of 5 mg/mL [165].

The application of microalgae-derived sulfated PSs was extensively evaluated for their ability to prevent the accumulation and activity of ROS and other radical stress agents [70,106]. Extracellular sulfated PSs have been largely utilized in cosmetic formulations to improve skin hydration and barrier function. Many companies have patented and commercialized these active ingredients in their products [166,167,168]. Their effectiveness as moisturizing agents in cosmetics is due to their strong water-retention capabilities, which align with their physiological role in microalgae to counteract desiccation [169]. As an example, a natural sulfated polysaccharide isolated from *Porphyridium* sp. is currently used in many cosmetic products [170], especially those developed in the Skin Actives Labs [171] and by Algologie [172]. Research on this PS has revealed a wealth of biological activities, demonstrating that it does not merely represent a physical barrier but provides active protection against photo damage and ageing [173]. Another example is Alguronic Acid^®^ from Algenist (San Francisco, CA, USA), an undetermined mix of polysaccharides produced by microalgae. The formulation used in this product showed significant anti-ageing properties, helping in the rejuvenation of the skin and a youthful appearance [174]. *Chlorella* is one of the main microalgal genera exploited for cosmetic products; its EPSs, obtained from heterotrophic growth, have been patented and commercialized in the formulation of anti-ageing products [175]. In addition, the study of *C. vulgaris* PS highlighted a reinforcement in collagen repair mechanisms [22]. Another kind of PS produced from microalgae and used in the cosmetic field is the ß-1,3-glucans; these PSs act as free radical collectors and are known to be active as anti-inflammatory compounds, as previously shown for those produced by fungi, plants (especially cereals), and microorganisms [31,176,177]. These molecules are used in cosmetic formulations as texturizing agents [178] and for their antioxidant and moisturizing properties, which help in maintaining skin integrity and protect from oxidative stress and inflammation, as shown for laminarin [179]. These PSs seem to be particularly suitable for treatment of reactive and sensitive skin types. Microalgal species like *Skeletonema costatum* (Bacillariophyta) [180] and *Porphyridium* sp. (Rodophyta) showed an interesting production of ß-1,3-glucans that seems to be promising [181,182].

Table 6 provides a list of microalgae species producing polysaccharides that have shown properties of potential interest for the cosmetic industry.

## 5. Concluding Remarks

**Figure 4:**
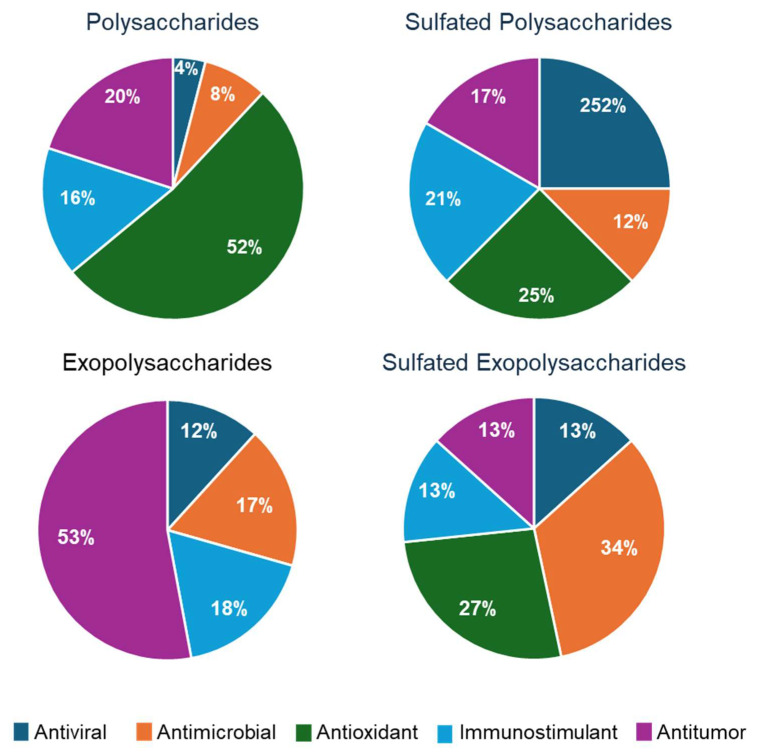
The pie diagrams show the percentage of diverse activities (see colour code) displayed by different classes of polysaccharides. Source data are those reported in Table 1, Table 2, Table 3, Table 4 and Table 5.

According to the studies reported here, microalgal polysaccharides present diverse biological activities of potential pharmaceutical interest. In particular, SP and EPS have been extensively studied in Rhodophyta, demonstrating bioactivity across several types of bioassays. SP produced by these microalgae showed strong antiviral, antimicrobial, immunostimulant, and anticancer activities, whereas EPSs were primarily active antimicrobial and antioxidant agents [182,183]. The most represented activity displayed by microalgae polysaccharides is antioxidant for PSs (Figure 4, in green) and antitumor for EPSs (Figure 4, in purple), while SPs present comparable hits for the different bioactivities evaluated. The type of activity reported more frequently for S-EPS is antimicrobial (34%, in orange), followed by antioxidant activity (24%, in green). It is interesting to note that, to the best of our knowledge, no hits for antioxidant activity have been reported for non-sulfated EPS.

These differences may rely on different properties of the polysaccharide classes, or may depend merely on the fact that some types of assays are of particular interest for identifying new possible hits. On the other hand, the correlation between the structure and the bioactivity is not always straightforward, although there is some evidence showing that the presence of sulfated groups, which can interact with cationic protein and macromolecules, appears to be a key characteristic influencing their biological effects [184]. In addition, research indicates that the biological activities of polysaccharides, such as immunomodulatory effects, are influenced by factors like glycosidic linkages (specifically β-(1→3) and β-(1→6) bonds), degree of branching, and chain length [159]. In the case of EPS, some studies suggest that their antimicrobial activity is linked to their ability to disrupt the bacterial cell envelope, in particular the peptidoglycan layer [182,183]. Other studies indicate that the polyanionic nature of EPSs contributes to their immunomodulatory and antiviral properties [185]. Additionally, the sugar composition of PSs, including the presence of uronic acids, carbohydrate acyl groups, and pyruvates, influences their anionic properties [186]. Overall, the effect of PS has been correlated to the size and degree of sulfation, the composition of the sugar units, the molecular weight, and the diversity of the linkage chemistry [68,72,73,75].

PSs used for cosmetic applications have been mainly found in Chlorophyta, Rhodophyta and Bacillariophyta. Their properties mainly rely on their ability to retain water, which makes them valuable for skincare formulations [187]. Notably, EPSs are promising candidates for topical applications, as slow-release moisturizing and protective agents, due to their viscosity, absorption capacity, and porosity [188].

The use of bioactive natural products presents, in general, several advantages over synthetic compounds, including lower toxicity, higher bioavailability, and greater sustainability. These benefits represent an additional value and have fuelled the interest in PSs for novel applications [189]. In this regard, microalgae represent a promising source of bioactive polysaccharides, as their controlled cultivation enables large-scale biomass production with relative ease [188], with a minimized impact on the environment, as demonstrated by the growing market interest in algae-derived polysaccharides, supported by investments in sustainable bioeconomy initiatives. This has led to a worldwide increase in biomass production for the extraction of bioactive molecules, with current microalgal biomass valued at approximately EUR 1000 per ton [190]. The production of PS from microalgae fits well in a Circular Bioeconomy Model. Indeed, microalgae can sequester CO_2_ from biogenic sources and utilize waste-derived nutrients, helping close resource loops. Microalgae cultivation may be integrated with anaerobic digestion, composting, and ethanol fermentation systems, which improves environmental and economic sustainability. In particular, the use of CO_2_ from ethanol fermentation is mostly advantageous, as the gas produced during the process is nearly pure (~99%), making it ideal for microalgae cultivation [191]. Most of the advancements in integrated cultivation systems towards zero-waste and circular economy approaches have been achieved only for a few eukaryotic microalgae species, e.g., those belonging to the genera *Chlorella*, *Dunaliella*, *Nannochloropsis*, *Scenedesmus* [192] and the model diatom *Phaeodactylum tricornutum* [193]. Despite the potential advantages of producing PSs from microalgae, only research on macroalgal bioactive polysaccharides has progressed further. Commercial applications and market permeation of microalgal PSs remain limited, with only a few microalgal PS-based products available on the market [194]. Challenges in their industrial exploitation primarily stem from difficulties in purification and structural characterization, as many studies focus only on bioactivity, without fully elucidating the chemical composition of PS fractions. This is partly due to the complexity of macromolecular structures and the high cost of specialized analytical instrumentation [195]. Additionally, PS production is highly dependent on culture conditions, which regulate the activation of biosynthetic genes. The choice of extraction method further influences the yield and purity of specific compounds. This variability complicates the standardization of procedures, making comparisons between studies difficult. On the other hand, diverse culture conditions and extraction techniques could also expand the potential for discovering novel bioactive molecules.

Recent studies tried to overcome some of these limitations, focusing on different aspects to optimize PS production. Lin et al. recently optimized the production of two SPs from the diatom *Hyalosynedra toxoneides*, acting first on culture conditions and then on the purification process. They applied a semi-continuous culture method wherein they varied the concentration of the medium, in particular by supplying macronutrients such as nitrogen, phosphorus and silicate and also vitamins and trace elements. Extraction and purification methods for SP were also optimized in terms of efficiency and purity, performing EtOH precipitation and deproteinization followed by anion-exchange chromatography using DEAE-Sephacel resins. This approach was performed in 8Lt custom-made photobioreactors and it enhanced the production of sulphated polysaccharides, reaching 2.46 mg/L/day in the optimal conditions [196].

As mentioned before, extraction is a crucial step to increase PS yield, and even now conventional and largely used methods involve hot-water, alkaline and acid extractions [197]. Different studies applied new approaches to improve the production yield of specific compounds using, for example, microwave-, ultrasound-, and enzyme-assisted procedures, pH shifting and pressurized liquid extraction, commonly used for macroalgae [17,198], fostering a more widespread use for microalgal biomass [199]. Interesting is the Response Surface Methodology (RSM) extraction method studied by Chen and coworkers (2024) for the red microalga *Porphyridium cruentum*, where a combination of different parameters was used to improve PS fraction yield; in this case, with an additional microwave-assisted extraction step, the PS production reached over 25% of DW [36]. A similar application was also performed for *Isochrisis galbana* to optimize PS yield [117]. RSM technique combines extraction time, temperature and a specific ratio between biomass and water; the conditions used are not standard and could be adapted for different species. This method presents different advantages like shorter extraction times, reduced costs, and use of water, which maintain structural characteristics of PS, not affecting activity [200].

Another interesting extraction approach is the one associated with the use of enzymes. This method helps to degrade the microalgal cell wall [201] and, in association with other extraction methods, could optimize polysaccharide yield. The choice of enzymes is linked to the different microalgal species considering their different cell-wall composition [59]. As an example, the use of enzymes like lysozymes and chitinases could be ideal for *Chlorella* sp. to attack N-acetylglucosamine polymer cell walls; as for *Nannochloropsis*, cellulase enzyme is ideal to efficiently extract β-glucans [202,203], while for *Chlamidomonas* sp., the best results are obtained with the use of α-amylase, proteases or lipases/phospholipases, according to cell-wall composition [204,205]. Peng and co-workers applied these advancements in the extraction of bioactive polysaccharides from *Chlorella* sp., where a microwaved-assisted approach and RSM were optimized by the use of a specific mixture of pectinase and cellulase enzyme [206]. The advantages of this technique are various: it helps the fragmentation of PS, simplifying structure and function studies, as well as considering that low molecular weights could increase bioactivity [72,146]; there is a decrease in extraction time and energy consumption; and it avoids the use of solvents, thus representing a green extraction method [207]. Another eco-friendly extraction method is the one mediated by the use of weak acids like citric or phosphoric acid as acid-catalyzed pre-treatment for microalgal biomass, which is especially useful for cell-wall polysaccharides [208]. This method grants the extraction of high concentrations of PS without any chemical removal or the use of hazardous solvents, just with a simple neutralization [209,210]. Further improvements may come from ultrasound and pulsed electric field (PEF) extraction [211], which disrupt cells non-thermally to release polysaccharides, as well as aqueous two-phase systems (ATPSs) for an eco-friendly, low-cost method for selective polysaccharide separation [212] that are already used to extract polysaccharides and other interesting compounds from plants.

Regarding polysaccharide characterization, classical approaches include Nuclear Magnetic Resonance (NMR) analytical techniques used to determine the structure, dynamics, reaction state, and chemical environment of the molecules. Mass Spectrometry (MS) with different ionization techniques (ESI, MALDI, SELDI) and analyzers (TOF, Ion Traps, quadrupoles, etc.) is also used, frequently associated with chromatography techniques (LC-MS, GC-MS) [213]. The latter are destructive methods, although over time they have become more sensitive and precise and, associated with other techniques, can produce optimized results [214]. Different approaches have been explored for non-destructive methods, like Raman micro-spectroscopy used for the study of single-cell chemical construction and of molecule production using laser light inelastic scattering. Through this approach, it has been possible to detect common signals for microalgal PSs in different microalgal species [213,215,216]. Another interesting non-destructive spectroscopic method for identification is Fourier-Transform InfraRed (FTIR) spectroscopy, which uses mid-infrared light absorption for detecting molecular groups and generating a unique fingerprint [59]. This approach is helpful when there is a change in the chemical composition of the microalgal cell wall, like in a study by Spain et al., who detected cell-wall polysaccharide changes in different Nordic microalgae (*C. vulgaris*, *H. pluvialis*, etc.) during their growth curve [217].

**Figure 5:**
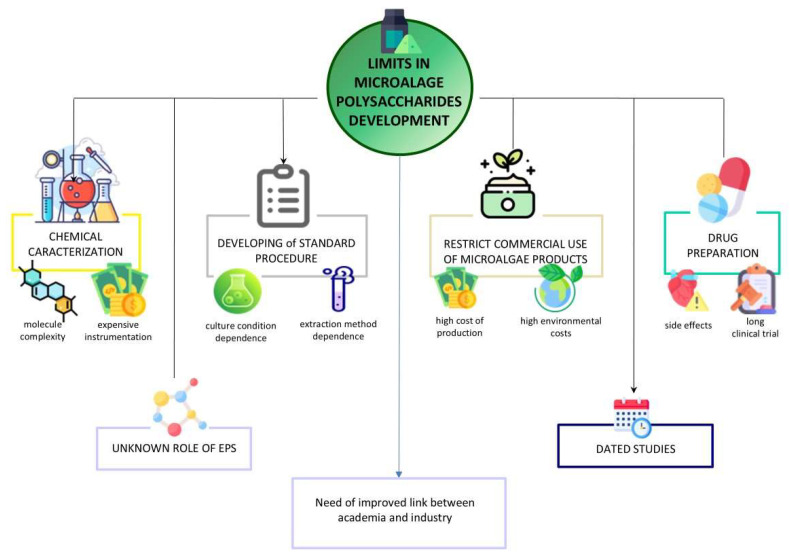
Schematic representation of the main factors limiting the development of microalgae polysaccharides at industrial level for biotechnological applications. Focus is given to limiting factors present in chemical characterization, the development of standardized procedures, microalgae commercialization and drug development.

Further obstacles arise in the pharmaceutical development of polysaccharides, including challenges associated with drug formulation and delivery, potential side effects [53], and the lengthy approval process required by regulatory authorities [189,218]. The main limitations hindering the development of microalgae polysaccharides are outlined in Figure 5.

To advance the exploitation of microalgae PSs for applications for human health, future research should focus on different aspects: further improving extraction methods, developing advanced analytical techniques for chemical characterization, and optimizing culture conditions to enhance standardization. There are several promising new technologies that can be used to overcome current challenges in implementing polysaccharide production from microalgae. Multi-omics platforms (genomics, transcriptomics, proteomics, metabolomics) are expected to greatly advance the exploitation of microalgae PSs as they will provide a better understanding of metabolic networks and identify bottlenecks in PS production. Possible solutions to low polysaccharide yield and high variability between strains may come from new genetic and metabolic engineering technologies, such as CRISPR/Cas9 and transcriptional activator-like effector nucleases (TALENs), which are now being applied to microalgae like *Chlamydomonas reinhardtii* [219,220] and *Nannochloropsis* [221,222]. This approach will enhance carbon flux toward polysaccharide synthesis and/or suppress competing pathways (like lipid accumulation). Synthetic biology platforms may also enable the introduction of novel biosynthetic pathways to produce polysaccharides with tailored properties.

To improve cultivation conditions and reduce high operational costs, it will be pivotal to develop AI-driven photobioreactors, which use real-time sensors and machine learning to optimize light, nutrients, and CO_2_, and biofilm-based cultivation to reduce water use and simplify harvesting. In addition, machine learning could be used to analyze large amounts of data from cultivation systems to predict optimal strain/environment combinations. Finally, 3D-printed microfluidic systems may enable rapid strain screening and optimization at a small scale before scaling up. Cascade biorefining approaches are increasingly recognized as a suitable way to improve the economic viability of production systems. This approach is based on the sequential extraction of high-value products (e.g., pigments → lipids → polysaccharides → protein → bioenergy). This approach, integrated with industrial CO_2_ emitters or the use of wastewater, may reduce input costs, improving sustainability [223]. Microalgae-based multiproduct biorefinery systems represent a strategically advanced approach to enhancing the techno-economic feasibility and environmental sustainability of biomass valorization processes. Sustainability metrics are further optimized through the integration of secondary resource streams, including nutrient-rich wastewater and CO_2_-laden flue gas, enabling nutrient recovery, carbon sequestration, and concomitant bioremediation [192]. This integrated utilization supports the establishment of a net-zero-emission, carbon-neutral production paradigm for the generation of high-value microalgal bioproducts and biomaterials.

Progress in these areas could facilitate drug development, particularly by addressing challenges associated with high-molecular-weight PSs, which may hinder cellular uptake and penetration into target cells. Additionally, further studies must focus on understanding the production and the role of EPSs to better exploit this source as bioactive molecules.

Overall, improving the sustainability of PS production, in terms of economic and environmental costs, could be the key to expand their use in the market and valorise the potential of microalgae.

## Figures and Tables

**Table 1:** List of species producing antiviral polysaccharides, classification of polysaccharides (EPS = exopolysaccharide, PS = polysaccharide, SP = sulfated polysaccharide, S-EPS = sulfated exopolysaccharide), monomeric composition, and target virus species.

Species	PS	Main Sugars	Target Virus	Active Concentration	Reference
*Porphiridium cruentum* *Tetraselmis suecica*	EPS	-	Viral Hemorrhagic Septicaemia virus (VHS)	100 µg/mL 100 µg/mL	[80]
*BTM 11*	PS	-	Hepatitis C-related virus	-	[81]
*Chlorella autotrophica*	SP	-	VHS African swine fever virus	47% of inhibition of virus replication at 2–20 µg/mL 67.4% of inhibition of virus replication at 20 µg/mL	[82]
*Cochlodinium polykrikoides*	SP	mannose, galactose, glucose	HIV1, HSV, RSVA-long Parainfluenza virus	1.7 µg/mL 4.5 µg/mL ^5^ 2–3 µg/mL 40–26.1 µg/mL	[79]
*Navicula directa*	SP	fucose, xylose, galactose, mannose, rhamnose	*Herpes simplex* 1 virus *Herpes simplex* 2 virus Influenza A virus	CC_50_/IC_50_ ^1^ = 270 CC_50_/IC_50_ = 510 CC_50_/IC_50_ = 32	[78]
*Porphyridium aerugineum*	SP	-	*Herpes simplex* 1 virus *Herpes simplex* 2 virus Varicella zoster virus Moloney murine sarcoma virus Moloney murine leukemia virus	CPE_50_ = 100 µg/mL CPE_50_ = 200 µg/mL CPE_50_ = 100 µg/mL Ffu_50_ ^3^ = 500 µg/mL RT_50_ ^4^ = 200 µg/mL	[74,77]
*Porphyridium* sp.	SP	xylose, glucose, galactose	*Herpes simplex* 1 virus *Herpes simplex* 2 virus Varicella zoster virus Herpes simplex 1 virus murine and rabbit model Moloney murine sarcoma virus Moloney murine leukemia virus	CPE_50_ ^2^ = 1 µg/mL CPE_50_ = 5 µg/mL CPE_50_ = 0.7 µg/mL 100 µg/mL 55% of recovered virus transforming capacity at 100 µg/mL 20% of recovered virus transforming capacity at 100 µg/mL	[74,76,77]
*Rhodella reticulata*	SP	-	*Herpes simplex* 1 virus *Herpes simplex* 2 virus Varicella zoster virus Moloney murine sarcoma virus Moloney murine leukemia virus	CPE_50_ = 10 µg/mL CPE_50_ = 20 µg/mL CPE_50_ = 8 µg/mL Ffu_50_ = 150 µg/mL RT_50_ = 50 µg/mL	[74,77]
*Gyrodinium impudicum*	S-EPS S-EPS	galactose galactose and uronic acid	Influenza virus type A Encephalomyocarditis virus	IC_50_ = 0.19–1.48 µg/mL IC_50_ = 26.9 µg/mL	[68,83]

Note: The table is organized in alphabetical order primarily according to the polysaccharide classification, and secondarily to species name. Abbreviations: ^1^ CC_50_/IC_50_: ratio between cytotoxicity value and IC_50_ value, both expressed as µg/mL. ^2^ CPE_50_: concentration of polysaccharide that offers 50% protection against the cytopathic effect. ^3^ Ffu_50_: concentration of polysaccharide that offers 50% protection against the formation of foci of malignant cells. ^4^ RT_50:_ the concentration of polysaccharide that offers 50% of reduction in virus reverse transcriptase activity. ^5^ The presence of two values is referred to, respectively, as polysaccharide A1 and A2.

**Table 2:** List of species producing antimicrobial polysaccharides, classification of polysaccharides (EPS = exopolysaccharide, PS = polysaccharide, SP = sulfated polysaccharide, S-EPS = sulfated exopolysaccharide), monomeric composition, and target microorganism species.

Species	PS	Main Sugars	Microorganism Targeted	Active Concentration	Reference
*Botryococcus braunii* *Chlorella pyrenoidosa*	EPS	–	*Escherichia coli* *Staphylococcus aureus*	MIC = 7.5 μg/mL MIC = 30 μg/mL	[94]
*Microchloropsis gaditana*	EPS	–	*Cladosporium cladosporioides*	500 mgGlcEq/L	[97]
*Rhodella reticulata*	EPS	–	*Staphylococcus aureus*	MIC = 250 μg/mL	[89]
*Chlorella pyreionodosa*	PS	glucose, galactose, rhamnose, mannose and arabinose, N-acetyl glucosamine, N-acetyl galactosamine	*Candida albicans* *Listeria monocytogenes*	0.1 mg–4 mg orally administered in mouse model	[93]
*Tetraselmis* spp.	PS	–	*Candida albicans* *Penicillium italic*	-	[96]
*Chlamydomonas reinhardtii*	SP	-	*Neisseria mucosa* *Escherichia coli* *Streptococcus* sp. *Bacillus subtilis*	MIC = 480 μg/mL MIC = 420 μg/mL MIC = 480 μg/mL MIC = 420 μg/mL	[98]
*Isochrysis galbana* *Nannochloropsis oculata*	SP	xylose, mannose, galactose, glucose, mannitol	*Pseudomonas aeruginosa* *Escherichia coli* *Candida krusei*	1870 µg/mL 1250 µg/mL and 2500 µg/mL 60 µg/mL and 80 µg/mL	[87]
*Porphyridium cruentum*	S-EPS	galactose, glucose	*Eschierichia coli* *Staphylococcus aureus* *Salmonella enteritidis*	1% of S-EPS aqueous solution	[88]
*Porphyridium marinum*	S-EPS	–	*Nosema ceranae*	100 μg/mL inhibit 90% of growth	[99]
*Neochloris oleobundan* *Phaeodactylum tricornutum* *Tetraselmis* sp.	S-EPS	–	*Helicobacter pylori*	50% of bacterial adhesion inhibition	[91]

Note: The table is organized in alphabetical order, primarily according to the polysaccharide classification, and secondarily to species name. MIC = minimum inhibitory concentration.

**Table 3:** List of species producing polysaccharides with antioxidant activity, classification of polysaccharides (PS = polysaccharide, SP = sulfated polysaccharide, S-EPS = sulfated exopolysaccharide), monomeric composition, and type of assay/activity.

Species	PS	Main Sugars	Assay/Activity	Reference
*Chlamydomonas* sp.	PS	mannitol, ribose, anhydrous glucose, xylose, fucose	In vivo protection against oxidative damage in mice model	[110]
*Chlorella pyrenoidosa*	PS	mannose, rhamnose, glucuronic acid, galacturonic acid, glucose, galactose, arabinose.	In vivo evaluation of antioxidant enzymes in *Caenorhabditis elegans*	[111]
*Chlorella pyrenoidosa*	PS	d-arabinose, d-glucose, d-xylose, d-galactose, d-mannose, rhamnose	DPPH ^1^, hydroxyl and superoxide anion radicals scavenging	[112]
*Chlorella* sp.	PS	galactose, arabinose, rhamnose, glucose	DPPH	[113]
*Chlorella vulgaris*	PS	rhamnose, ribose, arabinose, xylose, 2-deoxy-D-glucose, mannose, glucose, galactose, and glucosamine	In vivo evaluation of antioxidant enzymes in *Caenorhabditis elegans*	[114]
*Dictyosphaerium* sp.	PS	-	DPPH scavenging activities	[115]
*Dunaliella salina* *Scenedesmus obliquus*	PS	galactose, mannose, glucose, rhamnose	DPPH scavenging activities	[116]
*Isocrysis galbana*	PS (lambda-carrageenan) PS	galactose mannose, glucose, galactose, rhamnose	DPPH Hydroxyl and superoxide radicals scavenging ROS scavenging activity	[117,118]
*Odontella aurita*	PS (chrysolaminarin)	glucose	hydroxyl radicals scavenging	[109]
*Pavlova viridis* *Sarcinochrysis marina*	PS	uronic acid sulphate groups	Radical scavenging (LPO) ^2^ inhibition Mouse red blood cell hemolysis assay	[119]
*Rhodella reticulata*	PS	-	scavenging activities	[120]
*Chlamydomonas reinhardtii*	SP	-	DPPH, hydroxyl radicals scavenging	[121]
*Chlorella pyreinodosa*	SP	rhamnose, glucose, glucosamine, glucuronic acid, mannose, fucose, xylose, galactose, sulphate	In vitro hydroxyl and superoxide radicals scavenging activities in human intestinal Caco-2 cells	[122]
*Chlorella vulgaris*	SP	rhamnose, arabinose, fucose, xylose, mannose, glucose, galactose, glucosamine	In vitro in DPPH-, superoxide-, hydroxyl radical-scavenging activities, metal chelating ability; In vivo in *C. elegans* model, oxidative stress resistance, increasing of SOD and CAT activities	[123]
*Navicula* sp.	SP	glucose, rhamnose, galactose, mannose, xylose	DPPH free radical scavenging	[104]
*Rhodosorus* sp.	SP	glucose, galactose, xylose, galacturonic acid	DPPH and ABTS ^3^ radical scavenging activities	[124]
*Tribonema minus*	SP	rhamnose, arabinose, xylose, mannose, glucose galactose	DPPH, superoxide, hydroxyl radicals scavenging activities	[125]
*Botryococcus braunii*	S-EPS	arabinose, fucose, glucose, galactose	ABTS, hydroxyl, superoxidea nion, DPPH radical scavenging axtivities in vitro	[126]
*Chlorella* sp. *Chlorella sorokiniana* *Picochlorum* sp.	S-EPS	sucrose, glucose glucosamine	DPPH and ABTS radical scavenging activities	[103]

Note: The table is organized in alphabetical order, primarily according to the polysaccharide classification and secondarily to microalgae species name. ^1^ DPPH: 1,1-diphenyl-2-picrylhydrazyl free radical. ^2^ LPO: lipid peroxidation. ^3^ ABTS: 2,2′-azino-bis(3-ethylbenzothiazoline-6-sulfonic acid).

**Table 4:** List of species producing immunomodulant polysaccharides, classification of polysaccharides (EPS = exopolysaccharide, PS = polysaccharide, SP = sulfated polysaccharide, S-EPS = sulfated exopolysaccharide), monomeric composition, and main activity.

Species	PS	Main Sugars	Immunomodulant Activity	Reference
*Chlorella* sp.	EPS	glucosamine hydrochloride glucuronic acid	production of nitric oxide (NO), TNF-α and IL-6	[135]
*Neochloris oleoabundans*	EPS	glucose, mannose, galactose, xylose, ribose, arabinose, rhamnose	Reverse phagocytosis inhibition Enhancing lymphocyte proliferation	[136]
*Thraustochytriidae* sp.	EPS	-	Induction of B cell proliferation. Inhibition of IL-6 and INF-γ formation	[137]
*Chlorella ellipsoidea*	PS	rhamnose, mannose, galactose	Stimulation of NO and cytokine production in macrophages	[138]
*Dictyosphaerium chlorelloides*	PS	galactose, 2-O-methyl-galactse, rhamnose, mannose	Activation of NK and Tc, increase in IFN-γ expression	[139]
*Haematococcus lacustris*	PS	galactose, glucose, mannose	Stimulation of macrophage secretion of pro-inflammatory cytokine, TNF-α; enhance the expression of COX-2 and iNOS	[140]
*Phaeodactylum tricornutum*	PS	glucose (chrysolaminarin)	Increase in IL-1β, cytokine production, macrophage migration and phagocytosis activation	[133]
*Chlorella stigmatophora* *Phaeodactylum tricornutum*	SP		Enhancement of phagocytosis process	[132]
*Nannochloropsis oculata*	SP	glucose, mannose, rhamnose	Stimulation of murine ß-lymphocytes	[141]
*Tribonema* sp.	SP	glucose, galactose, xylose	Increase in macrophage cell viability and the production of IL-6, IL-10, TNF-α	[131]
*Porphyridium cruentum*	SP	ribose, fucose, xylose, arabinose, mannose, galactose, glucose, glucuronic acid	Stimulation of mice white blood cell differentiation and peritoneal macrophage profile modification	[39]
*Porphyridium cruentum*	S-EPS	-	Stimulation of macrophage proliferation and nitric oxide (NO) production	[128]
*Gyrodinium impudicum*	S-EPS	galactose	In vivo stimulation of macrophages and natural killer cells; production of IL-1b, TNF-a, and NO	[129]

Note: The table is organized in alphabetical order, primarily according to the polysaccharide classification and secondarily to microalgae species name.

**Table 5:** List of species producing potential antitumor polysaccharides, classification of polysaccharides (EPS = exopolysaccharide, PS = polysaccharide, SP = sulfated polysaccharide, S-EPS = sulfated exopolysaccharide), monomeric composition, and type of activity assessed.

Species	PS	Main Sugars	Activity	Reference
*Chlorella pyrenoidosa* *Chlorococcum* sp. *Scenedesmus* sp.	EPS	10 different monosaccharides	cytotoxic activity against colon cancer cell lines HCT116 and HCT8	[155]
*Chlorella vulgaris* *Chlorella zofingiensis*	EPS	10 different monosaccharides	cytotoxic activity against colon cancer cell line HCT8	[156]
*Gymnodinium* sp.	EPS	D-galactan sulphate, associated with L-(+)-lactic acid	DNA topoisomerase I and II inhibition in K562 cells	[154]
*Nitzschia palea*	EPS	–	apoptosis induction in A549 cells	[153]
*Parachlorella kessleri*	EPS	galactose, mannose, xylose, rhamnose, arabinose	in vivo antiproliferative activity against CT26 colon carcinoma in mouse model	[152]
*Thraustochytriidae* sp.	EPS	-	anti-proliferative activity against BG-1 ovarian, MCF-7 breast, and SW-620 colon cancer cell lines	[137]
*Chlamydomonas reinhardtii*	PS	α- and β-pyranoses containing furoic acid	anti-proliferative activity against colon cancer HCT-116, liver cancer HepG-2, and cervical cancer HeLa cells	[157]
*Chlorella pyrenoidosa*	PS	rhamnose, mannose, glucose, galactose	antitumor activity against A549	[158]
*Dunaliella salina*	PS	galactose, mannose, glucose, rhamnose	cytotoxic activity against HCT 116 cell line	[116]
*Isochrysis galbana*	PS	β-D-glucose	anti-proliferative on U937 human leukemic monocyte lymphoma cells	[159]
*P. tricornutum*	PS	xylose, fucose, glucose, glucuronic acid	blocking cell cycle and mitosis in HepG2 cells	[149]
*Chlamydomonas reinhardtii*	SP	-	anti-proliferative activity against breast cancer MDA-MB-231 cells	[121]
*Pavlova viridis*	SP	fructose, glucose, mannose, uronic acid	in vivo test in transplanted S180 mice	[150]
*Sarcinochrysis marina*	SP	arabinose, D-fructose and glucose	in vivo test in transplanted S180 mice	[151]
*Tribonema* sp.	SP	glucose, galactose, xylose	apoptosis induction, block of cell cycle and mitosis in HepG2 cells	[131]
*Porphyridium cruentum*	S-EPS	-	in vivo in transplanted S180 mice	[128]
*Porphyridium* sp.	S-EPS	-	inhibition of T-cell lymphoma line 24-1 proliferation Apoptosis induction in Graffi myeloid tumour	[147,148]

Note: The table is organized in alphabetical order, primarily according to the polysaccharide classification, and secondarily to microalgae species name.

**Table 6:** List of species producing polysaccharides for cosmetic applications, classification of polysaccharides (EPS = exopolysaccharide, PS = polysaccharide, SP = sulfated polysaccharide), monomeric composition, and cosmetic applications.

Species	PS	Main Sugars	Cosmetic Application	Reference
*C. vulgaris*	ESP	–	collagen repair mechanisms	[22]
*Porphyridium* sp.	ESP	–	inhibitor of hyaluronidase anti-allergic	[42]
*Chlorella* sp.	PS	–	anti-ageing	[175]
*Porphyridium* sp.	PS	β-1,3-glucans	moisturizing properties	[181,182]
*Skeletonema costatum*	PS	β-1,3-glucans	texturizing agents moisturizing properties	[180]
*P.cruentum*	SP	xylose, galactose and glucose	inhibitor of hyaluronidase and elastase	[165]

Note: The table is organized in alphabetical order, primarily according to the polysaccharide classification and secondarily to microalgae species name.

## Data Availability

No new data were created or analyzed in this study.

## Abbreviations

The following abbreviations are used in this manuscript:

EPS	exopolysaccharide
ESI	electrospray ionization
DEAE	diethylaminoethanol
GC-MS	gas chromatography-mass spectrometry
HIV	human immunodeficiency virus
HSV	herpes simplex virus
LC-MS	liquid chromatography-mass spectrometry
MALDI	matrix-assisted laser desorption/ionization
MLSV	measles virus
PS	polysaccharide
RSVA	respiratory syncytial virus type A
SELDI	surface-enhanced laser desorption/ionization
SP	sulfated polysaccharide
S-EPS	sulfated exopolysaccharide
TOF	time of flight
VHSV	haemorrhagic septicaemia virus
